# Dermatitis herpetiformis in an African woman

**DOI:** 10.11604/pamj.2018.30.119.14012

**Published:** 2018-06-12

**Authors:** Musonda Sharon Machona, Mehak Gupta, Victor Mudenda, Owen Ngalamika

**Affiliations:** 1 Dermatology and Venereology Clinic, Adult Hospital of University Teaching Hospital, Lusaka, Zambia; 2 Department of Pathology and Microbiology, Adult Hospital of University Teaching Hospital, Lusaka, Zambia

**Keywords:** Dermatitis herpetiformis, dietary gluten, autoimmune

## Abstract

Dermatitis herpetiformis (DH) is an autoimmune blistering disease of the skin. It is a result of hypersensitivity to dietary gluten. Diagnosis of DH can be challenging in a low prevalence, resource-limited population. We present the case of an African woman who presented with clinical features of DH.

## Introduction

Dermatitis herpetiformis (DH) is a cutaneous manifestation of gluten hypersensitivity. DH is not just a vesicobullous skin disease, but a cutaneous-intestinal disorder caused by hypersensitivity to gluten. Exposure to dietary gluten leads to formation of autoantibodies against intestinal self-antigens that cross-react with cutaneous autoantigens resulting in an inflammatory cascade that produces the skin lesions of DH. The lesions are vesicobullous, pruritic, and usually localized on elbows, knees and buttocks [1]. DH is known to mostly affect individuals of northern European origin and is considered to be rare in Asians and people of African descent [2]. It is associated with Celiac disease (CD) and usually regarded as a cutaneous manifestation of CD. It has a genetic predisposition and has been reported to affect more males than females [3]. One of the major clinical challenges is in differentiating DH from linear IgA bullous dermatosis, especially in a resource-limited setting where confirmation by direct immunofluorescence is not available. We present the case of an African woman who presented with clinical features suggestive of DH.

## Patient and observation

**Figure 1:**
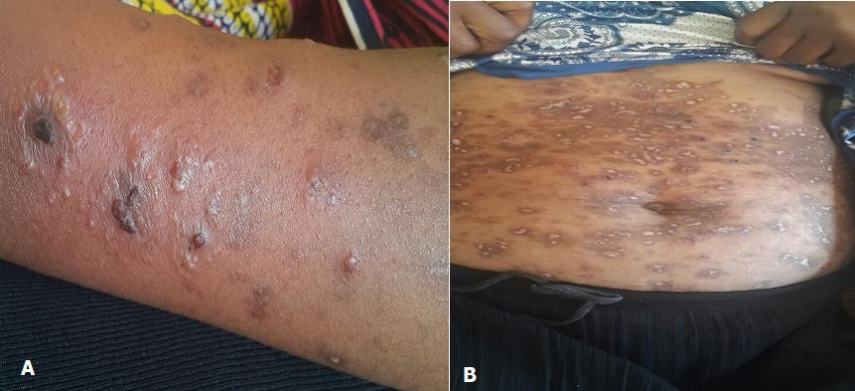
A) grouped vesicles on the forearm; B) healed lesions on the abdomen showing post inflammatory hypo- and hyper-pigmentation

**Figure 2:**
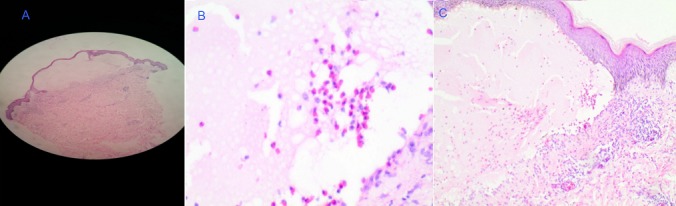
A) skin lesion showing sub-epidermal bulla and inflammation of dermis, H&E x40; B) eosinophils are seen within the bulla, H&E x400; C) picture showing the bulla and normal epidermis to one edge and the underlying mixed inflammatory infiltrate

A 30 year old female was referred to the University Teaching Hospital from a primary health centre for persistent itchy generalised body rash of 2 months. On admission, a diagnosis of acute gastritis with persistent skin infection was made. On subsequent review, the patient gave a history of the rash having started from upper limbs and then spread to the trunk and lower limbs. The rash begun as erythematous grouped vesicles preceded by an intense pruritus. The patient was also experiencing abdominal pains of two weeks' duration which were more severe during and after meals with associated nausea and vomiting. She denied taking any medications prior to development of the rash and had no previous history of any allergies. The review of other systems was unremarkable. She received intravenous and topical hydrocortisone at the referring clinic with no improvement noted. On examination, she was stable, not febrile and had no lesions in the oral cavity. The systemic examination was unremarkable. On local examination, she had generalised, symmetrical skin lesions in different stages of development (vesicles, erosions, crusts and post inflammatory hypo- and hyper-pigmented macules). The lesions were mostly affecting the trunk, buttocks, extensor surface of lower limbs and upper limbs, sparing the head, neck and palms. Vesicular lesions were grouped on an erythematous base (Figure 1). A diagnosis of dermatitis herpetiformis with suspected celiac disease was made. Endoscopy was done and a skin punch biopsy of a vesicle was taken and sent for histopathology which was suggestive of DH or Bullous Pemphigoid (Figure 2). The patient was started on Dapsone and a gluten-free diet. She improved markedly and was discharged with a planned follow up. The results of the investigations ordered are displayed in the table below (Table 1).

**Table 1:** Results of laboratory investigations done

TEST	RESULT
Serum IgA	High
Skin punch biopsy of intact vesicle	Intact sub-epidermal bulla seen with eosinophillic infiltration. Mixed cellular infiltrate centred on blood vessels seen in the dermis. Features suggestive of bullous pemphigoid or dermatitis herpetiformis
Liver function test	Normal
Renal function test	Normal
Endoscopy	Normal duodenum, ridge-like and healthy looking villi

## Discussion

DH is not a common condition in Zambia and therefore may not appear on the list of differential diagnoses of patients presenting with DH-like lesions. In a situation where an individual presents with atypical clinical features, the diagnosis of DH can be elusive [4]. However, our patient presented with typical clinical features of the disease. On the other hand, the histology of her skin lesions showed a subepidermal bulla with eosinophil instead of neutrophil infiltration which is more suggestive of bullous pemphigoid than DH. Therefore, correlating the clinical features to the histology picture was very important especially in the absence of direct immunofluorescence (DIF) which is the gold standard for diagnosis but was not available [5]. We aimed at performing serum IgA anti-epidermal transglutaminase in the absence of DIF. However, the lab could only do total serum IgA antibodies, which were elevated. IgA anti-epidermal transglutaminase could help in further supporting our diagnosis [6]. Serum IgA antibodies are the main autoantibodies produced against tissue transglutaminase in the gut and cross-react with epidermal transglutaminase in the skin leading to an inflammatory process resulting in DH lesions [3]. In the absence of DIF which is the gold standard for diagnosing DH and can accurately distinguish it from very similar conditions such as linear IgA bullous dermatosis, we used the clinical features and quick response to dapsone to support our diagnosis. However, linear IgA bullous disease could not be completely ruled out in our patient. As in most cases of DH in the absence of overt CD, the endoscopy done in our patient revealed a normal duodenum with healthy ridge-like villi. Furthermore, some patients with histologically severe CD may have normal-appearing mucosa at endoscopy [7]. Unfortunately, the biopsy specimens went missing and the patient was not agreeable to a repeat endoscopy. Therefore, we could not get a histology picture of her duodenal or jejunal mucosa. However, coeliac disease cannot be entirely ruled out in this patient based on endoscopy alone. The mainstays of DH treatment are a gluten free diet and dapsone. Both the enteropathy and cutaneous eruption are dependent on gluten ingestion. Hence a gluten free diet is a must for patients with DH. Upon diagnosis, dapsone is the drug of choice for patients until the gluten-free diet becomes effective [8]. On dapsone, symptoms quickly resolve within 2 to 3 days. Our patient improved markedly on dapsone and a gluten free diet. On subsequent reviews, the skin lesions had subsided leaving only post inflammatory dyspigmentation. The plan was to rechallenge the patient with a gluten diet after a few months and observe if the symptoms would recur. This would help in further ruling out linear IgA bullous dermatosis in our patient. However, the patient was subsequently lost to follow up.

## Conclusion

Although a number of specific laboratory tests for DH are currently not readily available in Zambia, this article is informative to clinicians on how to make a clinical diagnosis of DH with the aid of routine histopathology of intact vesicles. In addition, the typical histopathological picture of neutrophil infiltration may be absent, like in our patient, and therefore a clinicopathological correlation is important. Furthermore, this case is unusual because DH is known to be rare in people of African descent and is known to affect males more than females. More importantly, in the absence of DIF, which is the gold standard for diagnosis of DH, it is important to keep in mind the likelihood of linear IgA dermatosis as a possible diagnosis in such a patient.

## Competing interests

The authors declare no competing interests.
